# Robot-Assisted Abdominoperineal Resection for Anal Fistula Cancer in a Patient with Situs Inversus Totalis: A Case Report

**DOI:** 10.70352/scrj.cr.25-0557

**Published:** 2025-11-18

**Authors:** Yoshiaki Kita, Shinichiro Mori, Yusuke Tsuruta, Takuro Hirano, Shunya Iio, Asako Utsunomiya, Satoshi Iino, Kosei Maemura

**Affiliations:** Department of Digestive Surgery, Kagoshima City Hospital, Kagoshima, Kagoshima, Japan

**Keywords:** anal fistula cancer, rectal cancer, robot-assisted surgery, situs inversus totalis

## Abstract

**INTRODUCTION:**

Situs inversus totalis (SIT) is a rare congenital condition characterized by a complete mirror-image reversal of thoracic and abdominal organs. This anatomic anomaly poses technical challenges in abdominal surgery, particularly in oncologic procedures requiring precise orientation and dissection. Robot-assisted surgery (RS) offers advantages such as stable visualization and enhanced dexterity, which may facilitate safe and effective surgery even in patients with reversed anatomy. However, reports of using RS for colorectal cancer in the setting of SIT remain scarce.

**CASE PRESENTATION:**

A 75-year-old man was referred to our department for evaluation and management of an anal fistula. A comprehensive diagnostic workup revealed carcinoma with cutaneous invasion arising from the anal fistula in the context of SIT. Because of the cutaneous invasion, curative resection was indicated, and the patient underwent robot-assisted abdominoperineal resection (RAPR). To accommodate the mirror-image anatomy, the surgical setup—including the positioning of robotic arms, monitors, surgeon, assistant, and scrub nurse—was reversed relative to the standard configuration. Lymphadenectomy and vascular ligation were performed using a medial-to-lateral approach adapted in a left-right inverted fashion. While pelvic dissection and lateral mobilization were conducted using the surgeon’s right hand in a standard manner, cranial-side medial dissection and vascular handling were performed using the surgeon’s left hand after instrument exchange. The operative time was 334 min, there was minimal blood loss (15 mL), and the patient’s postoperative course was uneventful.

**CONCLUSIONS:**

RS is a feasible and safe option for patients with SIT undergoing colorectal cancer resection. This case highlights the adaptability of robotic platforms in facilitating unique techniques in the setting of complex anatomical variations. It supports their utility in achieving precise and complication-free oncologic surgery in rare scenarios. Further accumulation of similar patient reports is warranted to establish standardized strategies and validate outcomes.

## Abbreviations

BMI
: body mass index
CA19-9
: carbohydrate antigen 19-9
CEA
: carcinoembryonic antigen
IMA
: inferior mesenteric artery
LS
: laparoscopic surgery
RAPR
: robot-assisted abdominoperineal resection
RS
: robot-assisted surgery
SIT
: situs inversus totalis
UICC
: International Union Against Cancer

## INTRODUCTION

SIT is a rare congenital anomaly with an estimated incidence of approximately 1 in 5000 to 10000 individuals. In SIT, the thoracic and abdominal organs, along with the major vascular structures, are arranged in a complete mirror-image orientation relative to normal anatomy.^1)^ Although SIT itself does not typically affect organ function, the reversed anatomic configuration can present significant challenges during surgical procedures, particularly those requiring precise anatomic orientation.

Several reports have described the feasibility of laparoscopic colectomy in patients with SIT.^2,3)^ However, reports of RS for rectal cancer in this patient population remain scarce. The technical advantages of RS, including enhanced dexterity, 3D high-definition visualization, and tremor filtration, may outweigh the difficulties posed by the reversed anatomy.

In this report, we describe performing RAPR for anal fistula cancer in a patient with SIT, along with the surgical techniques and modifications employed to safely and effectively perform the procedure in the setting of this complex anatomic variation.

## CASE PRESENTATION

A 75-year-old man with a 20-year history of untreated anal fistula was referred to our hospital for evaluation and management. His medical history was notable for chronic kidney disease, which precluded the use of contrast agents during imaging. His height was 152.0 cm, weight 45 kg, and BMI 19.49 kg/m^2^. The serum tumor markers CEA and CA19-9 were within normal limits at 3.1 ng/mL and 15.9 U/mL, respectively.

**Fig. 1:**
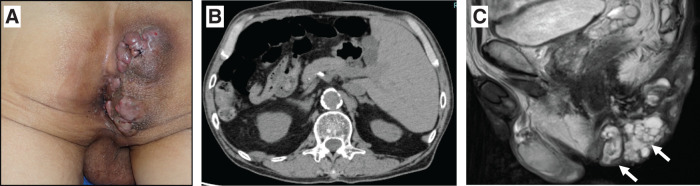
Preoperative findings. (**A**) Perianal region with cutaneous lesions. (**B**) CT revealed situs inversus totalis. (**C**) Primary region on MRI (arrow).

Physical examination revealed 2 elevated lesions with skin ulceration and mucous discharge on the right side of the perianal region (**Fig. 1A**). There were no remarkable findings on digital rectal examination or colonoscopy. CT revealed SIT (**Fig. 1B**), and MRI demonstrated that the primary lesion was located outside the rectal wall (**Fig. 1C**). Histopathologic examination confirmed the diagnosis of fistula-associated anal adenocarcinoma. Neither CT nor PET showed evidence of lymph node involvement or distant metastasis.

**Fig. 2:**
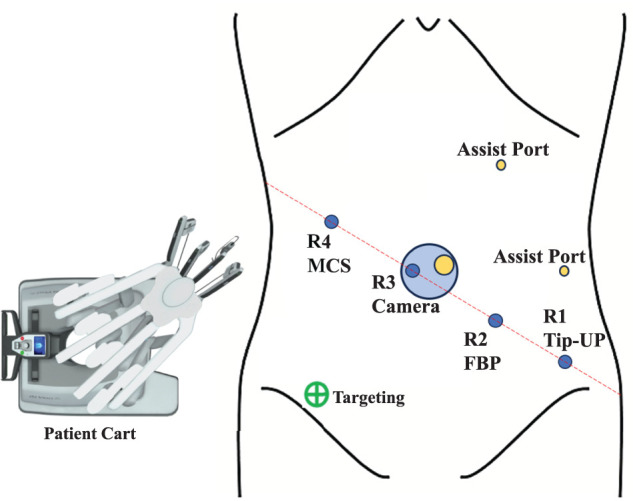
Port placement. FBP, fenestrated bipolar forceps; MCS, monopolar curved scissors; R1–4, robotic arms; Tip-UP, tip-up fenestrated grasper

Based on these findings, the patient was diagnosed with fistula-associated cancer, staged as cT3N0M0, cStage IIB according to the TNM classification for anal canal cancer (UICC 8th edition). We deemed surgical resection, including the area of skin invasion, to be appropriate and performed RAPR with lymph node dissection using the da Vinci Xi system (Intuitive Surgical, Sunnyvale, CA, USA). The patient cart was docked on the patient’s right side, and the 4 robotic ports were placed in a straight line from the left lower quadrant to the right upper quadrant, opposite to the usual configuration to accommodate the organ positioning of SIT. We made a midline incision and inserted an EZ Access port (Hakko, Nagano, Japan). Through this, we placed port number 3 for the robotic camera and the AirSeal port (ConMed, Largo, FL, USA) (**Fig. 2**). In total, we used 4 robotic ports and a 30° laparoscope.

**Fig. 3:**
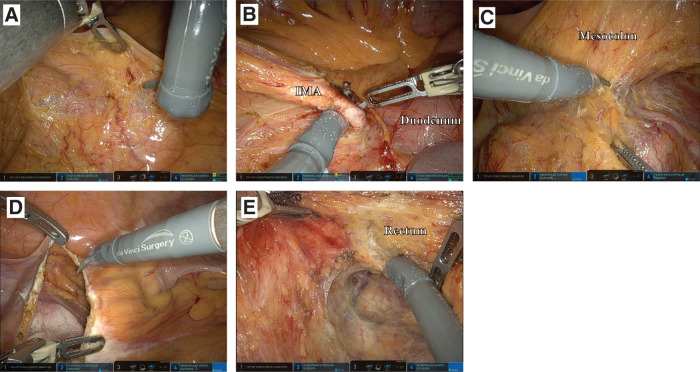
Intraoperative images. (**A**) Manipulation near the IMA. (**B**) Ligation of the IMA root. (**C**) Mobilization of the mesocolon. (**D**) Lateral approach. (**E**) Dissection of the rectum in the pelvic cavity. IMA, inferior mesenteric artery

There were no visible intra-abdominal adhesions. Because of the patient’s SIT anatomy, we performed the standard surgical procedure in a mirrored fashion. We initiated surgery with a peritoneal incision posterior to the IMA and performed ligation of the IMA with lymphadenectomy and mobilization of the colon using a medial-to-lateral approach. Initially, we used monopolar curved scissors with the right robotic arm (port number 4) and fenestrated bipolar forceps with the left arm (port number 2). However, manipulation with the right arm was restricted when working proximally near the IMA root (**Fig. 3A**). To overcome this, we switched the instruments: the fenestrated bipolar forceps were moved to the right arm (port number 4) and the monopolar curved scissors to the left arm (port number 2), allowing the procedure to continue smoothly (**Fig. 3B** and **3C**). We performed dissection in the pelvic cavity and distal rectal transection following standard techniques for RAPR (**Fig. 3D** and **3E**). Considering the possibility that the tumor might be more extensive than expected and involve the coccyx, we changed the patient’s position to the jackknife position after completing the robotic abdominal phase, in preparation for potential coccygectomy and wide skin reconstruction. After confirming the extent of the tumor using intraoperative ultrasonography, the perineal phase was performed. Because the lesion extended close to the right side of the coccyx, only skin reconstruction using a local flap was conducted to cover the defect.

The operative time was 334 min, excluding the cutaneous reconstruction, with an estimated blood loss of 15 mL. The patient’s postoperative course was uneventful, and he was discharged home on POD 14.

Pathologic examination confirmed fistula-associated adenocarcinoma measuring 80 × 60 mm with negative surgical margins and no lymph node metastasis.

## DISCUSSION

In recent years, favorable outcomes of RS for rectal cancer have been reported.^4–7)^ RS offers advantages such as motion scaling, high-definition 3D visualization, and the use of articulating instruments that enable precise manipulation. Although SIT itself does not affect life expectancy or organ function, the reversed anatomic orientation poses significant technical challenges during abdominal and pelvic surgeries. In the recent literature, an increasing number of case reports have described the successful use of RS in patients with SIT undergoing colorectal surgery. These reports suggest that RS can overcome some of the difficulties associated with reversed anatomy, facilitating safer and more effective procedures.^8–12)^ Here, we present our experience with RAPR for anal fistula cancer in a patient with SIT, highlighting the surgical strategy and technical considerations involved.

During surgery in the setting with SIT, simply mirroring the standard surgical procedure can help prevent misidentification of surgical anatomy and improve safety. However, in LS for patients with SIT, a simple mirror-image approach can be challenging due to the limited range of motion of straight instruments and the influence of the surgeon's handedness on port placement.^2)^ To perform surgery for SIT patients using the same approach as for normal anatomy, the surgeon must switch the roles of the right and left hands. This reversal can become a major obstacle because of the surgeon’s handedness. Some reports have suggested that LS in patients with SIT may be easier for left-handed surgeons.^13)^ Specifically, in LS for rectal resection with SIT, difficulty tends to arise during the dissection around the IMA, where the direction of dissection is nearly perpendicular to the direction of the straight instruments held in of the operator’s right hand. The articulating instruments of the robotic system are advantageous in this regard, as they can be flexed to the right, allowing the direction of movement to align with the intended dissection plane. Moreover, our switch technique further facilitates precise dissection in such situations by enabling optimal instrument positioning regardless of the surgeon’s handedness.

To date, there are only a few cases in the literature (5 in total) of RS for colorectal cancer in patients with SIT.^8–12)^ This case report is the 6th to describe RS for colorectal resection and the 5th to describe RS for rectal resection (**Table 1**). Although there are variations in port-placement strategies and surgical techniques across these reports, the safety and feasibility of RS for this type of surgery in patients with SIT have been consistently demonstrated. For the patient featured in this report, we used the da Vinci Xi system, with 4 ports aligned in a straight line from the left lower quadrant to the right upper quadrant, allowing for a single docking approach. Of particular note, we employed a unique switch technique in which the roles of the surgeon’s left and right hands were alternated during the procedure—a method not previously described in the literature. Although the articulating instruments of the robotic system are indeed useful, collisions between the dominant and retraction arms can occasionally occur during medial-to-lateral dissection of the left mesocolon before the inferior mesenteric vein division or during dissection around the lower rectum in the narrow pelvic cavity. Our unique “switch technique” may serve as an effective solution to overcome such collisions between the dominant and retraction arms within the confined working space of the pelvis.

**Table 1:** Previous reports of robot-assisted surgery for colorectal cancer in patients with situs inversus totalis

	Year	Author	Age/sex	Location	Operation	Operation time	Blood loss	Complications	Features
1	2012	Leong et al.^8)^	70/Female	Rectum	Low anterior resection	—	—	None	Square port positions
2	2015	Foo and Law^9)^	59/Male	Rectum	Low anterior resection	204	100	None	Mirror port positions
3	2018	Cui et al.^10)^	61/Male	Rectum	Low anterior resection	210	50	None	Square pors with Transanal NOSE
4	2020	Kasai et al.^11)^	60/Female	Rectum	Low anterior resection	194	Minimal	None	Dual docking
5	2024	Kato et al.^12)^	74/Female	Ascending colon	Hemicolectomy	218	5	None	Mirror port positions
6	2025	Our case	75/Male	Anal canal	Abdominoperineal resection	334	15	None	Mirror port positions

NOSE, natural orifice specimen extraction

Prior to the operation, a detailed preoperative simulation was conducted with the assistant surgeon, scrub nurse, and clinical engineer, who all adapted seamlessly to the mirror-setting. Although there have been no previous reports specifically addressing the assistant surgeon’s role in SIT cases, we consider that the burden on the assistant is reduced in RS compared with LS, as the robotic arms can replace much of the manual retraction and instrument handling with the assistant.

Despite its advantages, RS has certain limitations. First, because port-placement configurations vary depending on the specific robotic platform, it remains difficult to establish a universally applicable standard. Second, RS incurs higher costs than either open or conventional laparoscopic procedures, which may limit its widespread adoption.

We believe that RS using our unique switch technique can facilitate technically challenging procedures, allowing them to be performed safely and more easily and effectively.

## CONCLUSIONS

We report our experience of RAPR for anal fistula cancer in a patient with SIT. Our experience suggests that the surgeon’s adaptability, combined with the inherent flexibility of the robotic system, enables a safe and reliable surgical procedure. This switch technique may also contribute to overcoming the technical disadvantages of operating from the surgeon’s non-dominant side. Continued accumulation of similar case reports will be essential for establishing appropriate and standardized surgical techniques in the future.
